# IL-1β induces murine airway 5-HT_2A _receptor hyperresponsiveness via a non-transcriptional MAPK-dependent mechanism

**DOI:** 10.1186/1465-9921-8-29

**Published:** 2007-04-02

**Authors:** Yaping Zhang, Lars-Olaf Cardell, Mikael Adner

**Affiliations:** 1 Laboratory of Clinical and Experimental Allergy Research, Department of Otorhinolaryngology, Malmö University Hospital, Lund University, SE 205 02 Malmö, Sweden

## Abstract

**Background:**

Interleukin 1 beta (IL-1β) is found in bronchoalveolar lavage fluids from asthmatic patients and plays an important role in normal immunoregulatory processes but also in pathophysiological inflammatory responses. The present study was designed to investigate if IL-1β could be involved in the development of airway hyperresponsiveness and if transcriptional mechanisms, epithelium contractile factors and mitogen-activated protein kinase (MAPK) pathways are involved in IL-1β effect.

**Methods:**

The effect of IL-1β on 5-hydroxytryptamine (5-HT) induced bronchoconstriction was evaluated in an *in-vitro *model for assessment of long-term effects of inflammatory mediators on the airway smooth muscle. Murine tracheal segments were cultured up to 8 days in the absence or presence of IL-1β with subsequent evaluation in a myograph system, along with mRNA quantification, focusing on the role of the epithelium, acetylcholine release, transcriptional mechanisms and MAPK activity.

**Results:**

During control conditions, 5-HT induced a relatively weak contraction. Presence of IL-1β increased this response in a time- and concentration-dependent way. The increased concentration-effect curves could be shifted rightwards in a parallel manner by ketanserin, a selective 5-HT_2A _receptor antagonist, indicating that the responses are mediated by 5-HT_2A _receptors. The mRNA levels of 5-HT_2A _receptors were not changed as a consequence of the IL-1β treatment and actinomycin D, a general transcriptional inhibitor, failed to affect the contractile response, suggesting a non-transcriptional mechanism behind this phenomenon. Neither the removal of the epithelium nor the addition of atropine affected the IL-1β induced enhancement of 5-HT_2A _receptor-mediated contractile response. Application of inhibitors for c-Jun N-terminal kinase (JNK), p38 and extracellular signal-regulated kinase 1 and 2 (ERK1/2) showed that the signaling pathways for JNK and ERK1/2 dominated only in cultured segments (control) whereas JNK and p38 dominated in segments treated with IL-1β.

**Conclusion:**

IL-1β induces murine airway hyperresponsiveness, via a non-transcriptional up-regulation of 5-HT_2A _receptor-mediated contractile response. The increase of 5-HT contraction is unrelated to epithelial and cholinergic factors, but is dependent on IL-1β-induced changes of MAPK pathways. The fact that IL-1β can alter airway responses to contractile agents such as 5-HT, via alteration of the intracellular MAPK signal transduction pathways, might provide a new concept for future treatment of asthma.

## Background

5-hydroxytryptamine (5-HT) in the airways can be released from neuroendocrine cells, mast cells and platelets [1] and increased blood levels, correlating with the severity of asthma, have been reported [2]. 5-HT induces bronchoconstriction in most mammalian species via G-protein coupled receptors, termed 5-HT_2A _and 5-HT_1A _[3]. The former dominates clearly within murine airways [4], but information regarding the mediator pathways involved is somewhat contradicting. According to Moffatt and co-workers, 5-HT induces relatively weak contractions through epithelial 5-HT_2A _receptors via pathways involving muscarinic receptors [4], whereas Kummer and co-workers have reported the 5-HT_2A _receptors induce epithelium dependent contractions, unrelated to the muscarinic receptors, via a non-cholinergic contractile factor, in addition to a direct stimulatory effect on the smooth muscle [5].

Interleukin 1 beta (IL-1β) is one of the most pleiotropic and potent cytokines, produced predominantly by activated monocytes and macrophages [1,6]. It is known to play an important role in normal immunoregulatory processes but also in pathophysiological inflammatory responses [7,8]. Elevated levels of cytokines have been found in the bronchoalveolar lavage fluids from asthmatic patients [9], and the cytokines like tumor necrosis factor-alpha (TNF-α) and IL-1β have been shown to affect the airway smooth muscle response to various contractile agonists, like bradykinin, methacholine and 5-HT [10,11]. We have recently demonstrated that TNF-α induces a transcription dependent up-regulation of bradykinin receptors in the airway smooth muscle, resulting in an increased contractile response. This up-regulation appears to be dependent on mitogen-activated protein kinase (MAPK) pathways, like c-Jun N-terminal kinase (JNK) and extracellular signal-regulated kinase 1 and 2 (ERK1/2) [12,13]. However, we have also demonstrated that the same MAPK pathways are involved in IL-1β induced down-regulation of endothelin B receptor-mediated airway contractions [14]. Thus, the present study was designed to investigate if IL-1β affects 5-HT induced airway contraction focusing on the roles of transcription and MAPK activity and epithelium contractile factor. We have demonstrated that IL-1β induces 5-HT_2A _receptor-mediated hyperresponsiveness in the airway through MAPK activation, non-transcriptional and epithelium-independent mechanisms.

## Methods

### Tissue preparation

10 weeks old male BALB/c J mice (MB A/S, Ry, Denmark) were sacrificed by cervical dislocation, the whole trachea was rapidly removed and placed into Dulbecco's Modified Eagle's Medium (DMEM, 4500 mg/l D-glucose, 110 mg/l sodium pyruvate, 584 mg/l L-glutamine), supplemented with penicillin (100 U/ml) and streptomycin (100 μg/ml). The trachea was then dissected free of adhering tissue under a microscope and cut into three to four segments for subsequent organ culture. The experimental protocols have been approved by Lund University Animal Ethics Committee (M232-03).

### Organ culture

After the dissection, the segments were placed individually into wells of a 96-well plate (Ultra-low attachment; Sigma, St. Louis, MO, U.S.A.) with 300 μl serum free DMEM culture medium incubated at 37°C in humidified 5% CO_2 _in air in the absence and presence of recombinant murine IL-1β for the required time intervals (1, 2, 4 or 8 days). Segments were transferred into new wells containing fresh media including IL-1β every day.

### Epithelium removal

The epithelium of trachea was removed by gentle perfusion with 0.1% Triton X-100 for 1 min [4]. The epithelium was removed after the trachea had been cultured in the absence and presence of IL-1β. The removal of epithelium was verified by the absence of response to bradykinin in segments pre-constricted by carbachol (1 μM).

### In-vitro pharmacology

The cultured segments were immersed in temperature-controlled (37°C) myograph bath (Organ Bath Model 700MO, J.P. Trading, Aarhus, Denmark) containing 5 ml Krebs-Henseleit buffer solution (143 mM Na^+^, 5.9 mM K^+^, 1.5 mM Ca^2+^, 2.5 mM Mg^2+^, 128 mM Cl^-^, 1.2 mM H_2_PO_4_^2-^, 1.2 mM SO_4_^2-^, 25 mM HCO_3_^- ^and 10 mM D-glucose). The solution was continuously equilibrated with 5% CO_2 _in O_2 _to result in a stable pH of 7.4. Each tracheal segment was mounted on two L-shaped metal prongs. One prong was connected to a force-displacement transducer for continuous recording of isometric tension by the Chart software (AD Instruments Ltd., Hastings, U.K.). The other prong was connected to a displacement device, allowing adjustment of the distance between the two parallel prongs. Following equilibration, a pre-tension about 0.8 mN was applied to each segment and adjusted to this level of tension for at least one hour [15]. Each segment was then contracted with 60 mM KCl to test the contractile function. To inhibit epithelial prostaglandin release, the segments were incubated with 3 μM indomethacin 30 min before administration of 5-HT. At the end of the experiments, a reference contraction of 1 mM carbachol was induced.

### Data Analysis

All data are expressed as mean values ± S.E.M. Contractile responses to 5-HT in each segment were expressed as percent of maximal contraction induced by 1 mM carbachol (% of Cch) or the absolute values (mN) in the experiments with actinomycin D, atropine and three MAPK inhibitors, since they affect the reference (carbachol)-induced contraction. Each agonist concentration-effect curve was fitted to the Hill equation using an iterative, least square method (GraphPad Prism 4, San Diego, U.S.A), to provide estimates of maximal contraction (E_max_) and pEC_50 _values (negative logarithm of the agonist concentration that produces 50% of the maximal effect). Two-way analysis of variance (ANOVA) with Bonferroni post-test was used to compare the two corresponding data points at each concentration of the two curves, and unpaired Students' *t*-test with Welch's correction applied for the comparison of pEC_50 _values (curve shift) and E_max_. The data and statistical analysis was performed by GraphPad Prism 4 (San Diego, USA). P < 0.05 was considered as statistically significant.

### Chemicals

Recombinant murine IL-1β (R&D Systems, Abingdon, U.K); SP600125 (anthrax (1,9-cd)pyrazol-6(2H)-one, Calbiochem, Bad Sodem, Germany), SB203580 (4-[5-(4-Fluorophenyl)-2-[4-(methylsulfonyl)phenyl]-1*H*-imidazol-4-yl]pyridine, Tocris-Cookson, Bristol, U.K), penicillin and streptomycin (Life Technologies, Gathisburg, U.S.A.), PD98059 (2-(2-amino-3-methoxyphenyl)-4H-1-benzopyran-4-one), 5-hydroxytryptamine, ketanserin, atropine, indomethacin, carbachol, dimethyl sulfoxide (DMSO), KCl, Triton X-100, DMEM and Krebs-Henseleit Buffer (Sigma, St. Louis, MO, U.S.A.).

### mRNA study

Tracheal smooth muscle was isolated mechanically on an ice tray under a microscope. The samples with and without epithelium were stored in the RNAlater™ (QIAGEN GmbH, Hilden, Germany) under -80°C until use for extraction of total RNA. The tissues were homogenized and the total RNA was extracted by using the RNeasy Mini kit following the supplier's instructions (QIAGEN GmbH, Hilden, Germany). The purity of total RNA was checked by a spectrophotometer and the wavelength absorption ratio (260/280 nm) was between 1.6 and 2.0 in all preparations. Reverse transcription of total RNA (0.3–0.4 μg) to cDNA was carried out using Omniscript™ reverse transcriptase kit (QIAGEN GmbH, Hilden, Germany) in 20 μl volume reaction at 37°C for 1 h by using Mastercycler personal PCR machine (Eppendorf AG, Hamburg, Germany).

Specific primers for the mouse 5-HT_2A _receptor, choline acetyltransferase (ChAT) and house keeping gene β-actin were designed by using Prime Express^® ^2.0 software (Applied Biosystem, Forster city, CA, USA) and synthesized by DNA Technology A/S (Aarhus, Denmark). Sequences as follows:

5-HT_2A _receptor (GenBank: NM_172812):

Forward: 5'-GGG CCA AAT TAT CCT CCT TCA-3'

Reverse: 5'-ATC GTC CTT CGG CCT GC-3'

ChAT (GenBank: NM_009891):

Forward: 5'-CCT GGA TGG TCC AGG CAC T-3'

Reverse: 5'-GTC ATA CCA ACG ATT CGC TCC-3'

β-actin (GenBank; NM_007393)

Forward: 5'-TGG GTC AGA AGG ACT CCT ATG TG-3'

Reverse: 5'-CGT CCC AGT TGG TAA CAA TGC-3'

Real-time polymerase chain reaction (real-time PCR) was performed with the QuantiTect™ SYBR^® ^Green PCR kit (QIAGEN GmbH, Hilden, Germany) in The Smart Cycler^® ^II system (Cepheid, Sunnyvale, CA, USA). The system automatically monitors the binding of a fluorescent dye SYBR^® ^Green to double-stranded DNA by real-time detection of the fluorescence during each cycle of PCR amplification. The real-time PCR was prepared in 25 μl reaction volumes and carried out with heating 95°C for 15 min followed by touch down PCR i.e. denature at 94°C for 30 sec and annealing at 66°C for 1 min for the first PCR cycle, thereafter, a decrease of 2°C for the annealing temperature in every cycle until 56°C. Finally, 40 thermal cycles with 94°C for 30 sec and 55°C for 1 min were performed. The data were analysed with the threshold cycle (C_T_) method and the specificity of the PCR products was checked by the dissociation curves and visualized by agarose electrophoresis. Expected PCR products of 5-HT_2A _receptor 111 bp, ChAT 102 bp and β-actin 102 bp with a single band for each product were seen. A blank (no template) was included in all the experiments for negative controls.

The house keeping gene β-actin mRNA is continuously expressed to a constant amount in the cells. We compared its expression with expression to another house keeping gene GAPDH in a pilot study by real-time PCR and found no difference in the expression between the two house keeping genes. β-actin was used as a reference in this study, but both were equally constant in the tests. The relative amount of mRNA was obtained by the C_T _values of mRNA for 5-HT_2A _receptor or ChAT in relation to the C_T _values of mRNA for house keeping gene β-actin in the same sample by the formula *X*_0_/*R*_0 _= 2^*CtR*-*CtX*^, where *X*_0 _is the original amount of target mRNA, *R*_0 _is the original amount of β-actin mRNAs, *CtR *is the *C*_*T *_value for β-actin mRNAs, and *C*_*T*_*X *is the *C*_*T *_value for the target. Data are presented as mean ± S.E.M. Statistical analyses were used with unpaired Students' *t*-test with Welch's correction. *P *< 0.05 was considered to be significant.

## Results

### Up-regulation of 5-HT-induced contraction

**Figure 1:**
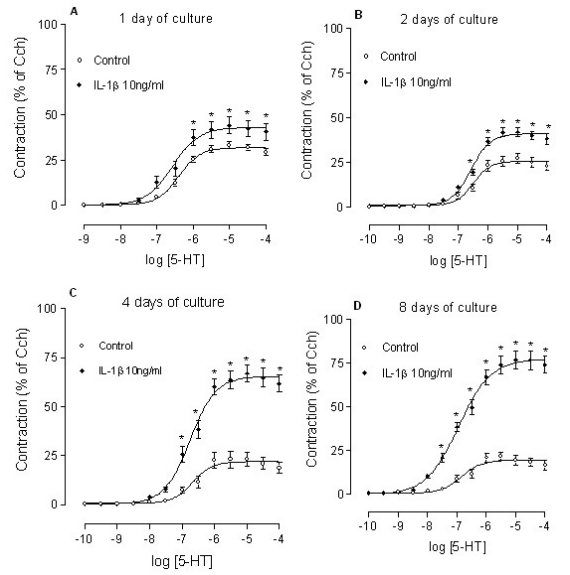
**Time-course for IL-1β effects on the 5-HT response**. Tracheal segments cultured for 1 day (A), 2 days (B), 4 days (C) or 8 days (D) in the absence (control) and presence of IL-1β (10 ng/ml). Statistical analysis was performed with two-way ANOVA with Bonferroni post-test to compare the two corresponding data points at each concentration of the two curves. Each data point is derived from 6–18 experiments and represented as mean ± S.E.M. *P < 0.05, compared with control.

**Figure 2:**
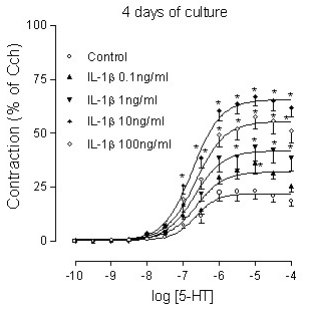
**Concentration-response curves for IL-1β effects on the 5-HT response**. Tracheal segments cultured for 4 days in the presence of different concentrations of IL-1β (0.1, 1, 10, 100 ng/ml). Each data point is derived from 6–16 experiments and represented as the mean ± S.E.M. Statistical analysis was performed with two-way ANOVA with Bonferroni post-test to compare the two corresponding data points at each concentration of the two curves. *P < 0.05, compared with control.

Tracheal segments were cultured in the absence and presence of IL-1β (10 ng/ml) for 1, 2, 4 or 8 days. A significant increase of the maximal contractile response to 5-HT was seen at 1 day, and this up-regulation became further enhanced when the culture periods were extended. The pEC_50 _values remained unaltered over time (Fig. 1A–D). When tracheal segments were cultured for 4 days in the presence of different concentrations of IL-1β (0.1, 1, 10 or 100 ng/ml), the 5-HT response increased in a concentration-dependent way. IL-1β concentrations higher than 10 ng/ml did not further increase the contractions. The pEC_50 _values remained the same in all groups (Fig. 2).

IL-1β (10 ng/ml) did not affect carbachol-induced maximal contraction in the segments cultured for 1 day (control E_max _= 7.9 ± 0.7 vs. IL-1β E_max _= 7.1 ± 1.0 mN, n = 6), 2 days (control E_max _= 6.5 ± 0.7 vs. IL-1β E_max _= 6.6 ± 0.7 mN, n = 7), 4 days (control E_max _= 6.1 ± 0.4 vs. IL-1β E_max _= 6.3 ± 1.0 mN, n = 6) and 8 days (control E_max _= 3.6 ± 0.5 vs. IL-1β E_max _= 3.5 ± 0.5 mN, n = 12).

### Pharmacological characterization of 5-HT receptors

**Figure 3:**
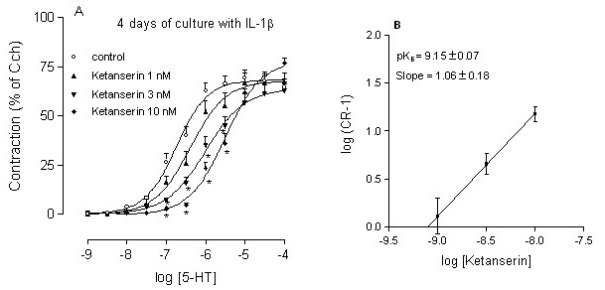
**Receptor characterization**. Effect of the selective 5-HT_2A _receptor antagonist, ketanserin (1–10 nM) on 5-HT induced contraction in the tracheal segments cultured for 4 days with IL-1β (10 ng/ml) (A). Ketanserin was added to the tissue bath 30 min before the first concentration of 5-HT. Each data point is derived from 5–13 experiments and represented as the mean ± S.E.M. Statistical analysis was performed with two-way ANOVA with Bonferroni post-test to compare the two corresponding data points at each concentration of the two curves (Fig. 3A), and unpaired Students' *t*-test with Welch's correction applied for the comparison of pEC_50 _values (curve shift) and the E_max _(see results). *P < 0.05, compared with control. Schild analysis of data derived from panel A (B).

The contractile response curves for 5-HT in the segments cultured for 4 days with IL-1β (10 ng/ml) were shifted to the right in a parallel manner by ketanserin (a selective 5-HT_2A _receptor antagonist) in the concentration of 1, 3 and 10 nM. The control pEC_50 _value (6.71 ± 0.05) in comparison with ketanserin at 1 nM pEC_50 _(6.37 ± 0.09, n = 7–13, P < 0.05), at 3 nM pEC_50 _(5.96 ± 0.09, n = 7–13, P < 0.001) and at 10 nM pEC_50 _(5.52 ± 0.07, n = 5–13, P < 0.001) were significantly higher (Fig. 3A), while the maximal contraction was not affected (control E_max _68.4 ± 3.9% vs. ketanserin at 1 nM E_max _67.8 ± 4.8%, at 3 nM E_max _64.6 ± 3.8 and at 10 nM E_max _77.1 ± 2.9%, n = 5–13, P > 0.05, Fig. 3A). A pK_B _value of 9.15 indicated that these contractions were mediated mainly via 5-HT_2A _receptors (Fig. 3B) in accordance with our previous results [15].

### Role of transcription

**Figure 4:**
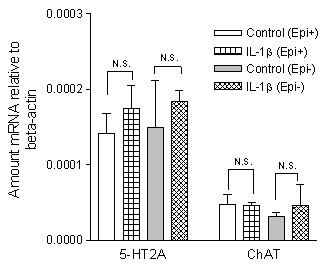
**mRNA expression for 5-HT_2A _receptors and choline acetyltransferase (ChAT)**. The trachea cultured in the absence (control) and presence of IL-1β (10 ng/ml). A 2-day culture period was used for experiments with intact epithelium and 4 days period for denuded segments. Each data point is derived from 2–5 experiments. Each value was derived from 3 mice and presented as mean ± S.E.M. Unpaired student's *t-*test with Welch's correction were used for statistic analysis. N.S. = not significant. Epi^+ ^= with epithelium, Epi^- ^= without epithelium.

**Figure 5:**
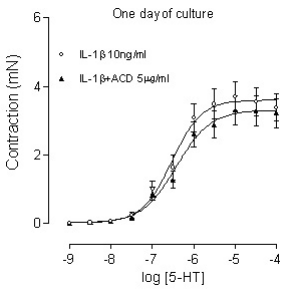
**Effect of actinomycin D on the IL-1β enhanced 5-HT induced contraction**. The tracheal segments were cultured for 1 day in the presence of IL-1β (10 ng/ml) with and without actinomycin D (ACD, 5 μg/ml). Each data point is derived from 7–18 experiments and represented as the mean ± S.E.M. Statistical analysis was performed with two-way ANOVA with Bonferroni post-test to compare the two corresponding data points at each concentration of the two curves, no significant differences were seen (P > 0.05).

The mRNA expression levels for the 5-HT_2A _receptors were not affected by the presence of IL-1β (Fig. 4). Neither was the contractile response induced by 5-HT after 1 day in culture with IL-1β (10 ng/ml) affected by the presence of actinomycin D (5 μg/ml), a general transcriptional inhibitor (P > 0.05, Fig. 5). Since actinomycin D affected the contraction induced by the reference substance carbachol (data not shown), the values are expressed in mN. Together these experiments indicate that the increased 5-HT_2A _receptor activity seen as the result of IL-1β pretreatment appears to take place without changes at the transcriptional level.

### Role of acetylcholine

**Figure 6:**
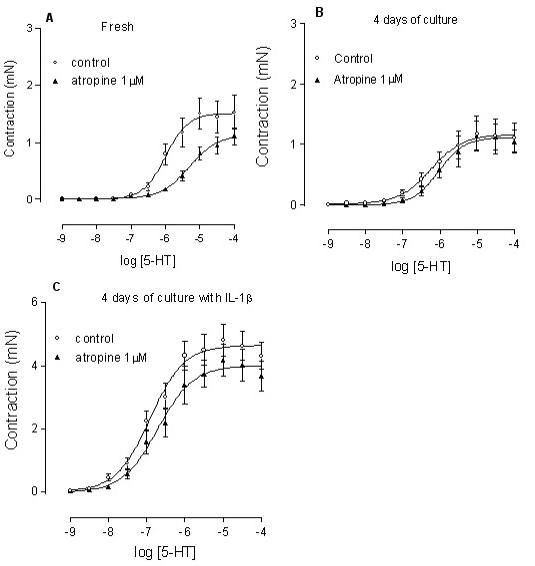
**Effect of atropine on 5-HT-induced contraction**. Atropine (1 μM) was added 1 hour before administration of the 5-HT concentration effect curves. Fresh (A), 4 days of organ culture in the absence (B) and presence (C) of IL-1β (10 ng/ml). Each data point is derived from 6–11 experiments and represented as the mean ± S.E.M. Statistical analysis was performed with two-way ANOVA with Bonferroni post-test to compare the two corresponding data points at each concentration, no differences were seen (Fig. 6A-C, P > 0.05). In addition, unpaired Students' *t*-test with Welch's correction was applied for comparison of pEC_50 _values (curve shift) and maximal contraction of the two corresponding curves (see results).

Choline acetyltransferase (ChAT) is responsible for acetylcholine production in all types of cell, including airway epithelium, smooth muscle and neuronal tissues [16,17]. The mRNA levels of ChAT were similar in tracheal segments cultured for 4 days regardless of the eventual presence of IL-1β (Fig. 4). Atropine (1 μM) was added 1 hour before administration of 5-HT [5,18]. In the fresh segments, atropine shifted 5-HT concentration response curves to the right (control pEC_50 _5.92 ± 0.07 vs. atropine pEC_50 _5.21 ± 0.07, n = 7–13, P < 0.001, Fig. 6A) with a tendency of decrease in the maximal contraction (control E_max _1.53 ± 0.28 vs. atropine E_max _1.18 ± 0.13 mN, n = 7–13, P > 0.05, Fig. 6A). However, after the segments were cultured for 4 days, both in absence and presence of IL-1β, the contraction induced by 5-HT became atropine insensitive i.e. no significant shifts of curves were seen after application of atropine (P > 0.05, Fig. 6B–C).

### Effects of epithelial removal

**Figure 7:**
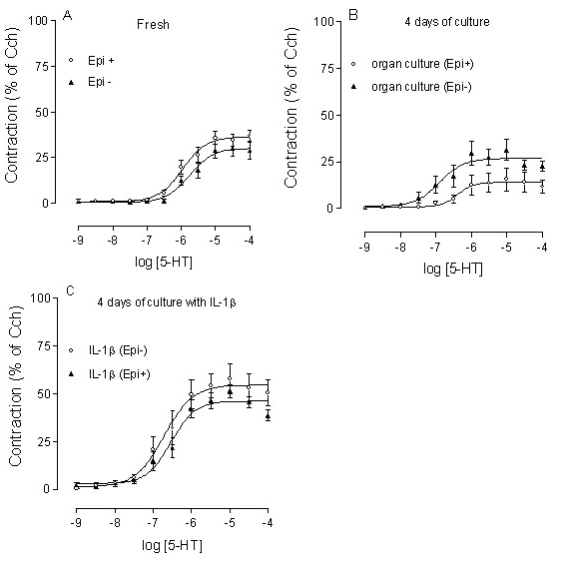
**Effect of epithelium removal on 5-HT-induced contraction**. Fresh (A), 4 days of organ culture in the absence (B) and presence (C) of IL-1β (10 ng/ml). Epithelium was removed after the trachea had been organ cultured in the absence and presence of IL-1β. The results were compared with data obtained in segments with an intact epithelium. Each data point is derived from 5–10 experiments and represented as the mean ± S.E.M. Statistical analysis was performed with two-way ANOVA with Bonferroni post-test to compare the two corresponding data points at each concentration, no significant differences were seen (P > 0.05).

In order to ascertain if epithelium is involved in the up-regulation of 5-HT-induced contraction, the epithelium was removed after the trachea had been cultured for 4 days in the absence and presence of IL-1β (10 ng/ml). Removal of epithelium did not affect the contractile response to 5-HT in the fresh segments (P > 0.05, Fig. 7A) and after treatment with IL-1β (P > 0.05, Fig. 7C), whereas a not statistical significant increase was seen after organ culture (P > 0.05, Fig. 7B). Neither was the mRNA level of 5-HT_2A _receptors nor the amount of ChAT affected by this procedure (Fig. 4). This excludes a role for epithelial factors in the IL-1β induced 5-HT hyperresponsiveness.

### Mitogen-activated protein kinase inhibition

**Figure 8:**
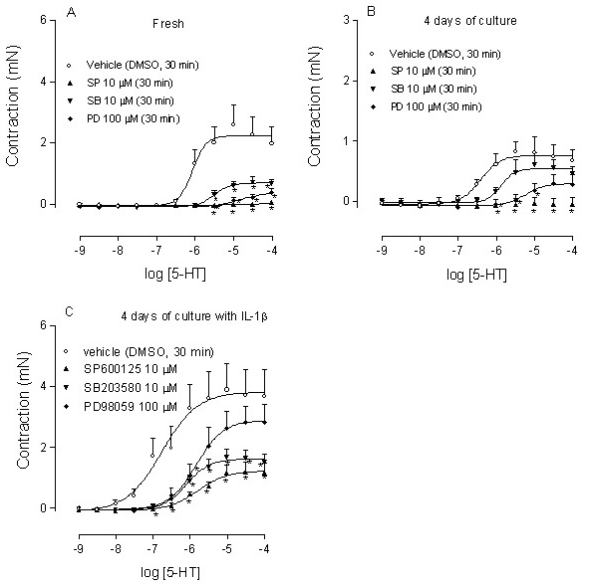
**MAPK inhibition**. Effect of specific inhibitors for JNK (SP600125), ERK 1/2 (PD98059) or p38 (SB203580) on 5-HT induced contractions. Fresh (A), 4 days of organ culture in the absence (B) and presence (C) of IL-1β (10 ng/ml). The three inhibitors as well as the vehicle (DMSO) were added to the tissue bath 30 min before the first concentration of 5-HT. Each data point is derived from 5–6 experiments and represented as mean ± S.E.M. Statistical analysis was performed with two-way ANOVA with Bonferroni post-test to compare the two corresponding data points at each concentration of the two curves. *P < 0.05, compared with control.

In order to investigate whether intracellular JNK, p38 and ERK1/2 were involved in the IL-1β induced up-regulation of the 5-HT response, a series of experiments were performed by use of the MAPK inhibitors SP600125 (10 μM), SB203580 (10 μM) and PD98059 (100 μM), and vehicle (DMSO) as control. The inhibitors were applied 30 min prior to the first 5-HT concentration in either fresh segments or segments cultured for 4 days in the absence and presence of IL-1β (10 ng/ml). In the fresh segments, 5-HT-induced contraction was almost completely abolished by SP600125, SB203580 and PD98059, with a reduction by 98%, 68% and 82%, respectively (Fig. 8A). Segments cultured in the absence of IL-1β had a similar inhibitory pattern (85% by SP600125, 21% by SB203580 and 60% by PD98059, respectively) as the fresh segments, although SB203580 had significantly less inhibitory effects (Fig. 8B). In segments treated with IL-1β, the inhibitory pattern was altered (68% in SP600125, 56% in SB203580 and 22% in PD98059, respectively), which indicates that the signalling was shifted from ERK1/2 to p38 in 5-HT-induced contraction (Fig. 8C). Taken together these data indicate that MAPK activities are required for 5-HT_2A _receptors-mediated contraction and that IL-1β induces an increased contraction to 5-HT by interference with MAPK pathways.

## Discussion

The present study demonstrates that long-term exposure of IL-1β increased the contractile response to 5-HT reflecting the development of airway hyperresponsiveness. Experiments with ketanserin indicated that the contraction was mediated via 5-HT_2A _receptors and since epithelial removal did not affect the outcome, these receptors must be situated direct on the airway smooth muscle. The mRNA levels of the 5-HT_2A _receptors were not changed as a consequence of the IL-1β treatment which suggests that these events might be regulated on a post-transcriptional level, an argument further supported by the failure of actinomycin D to affect the outcome. The experiments with a set of specific JNK, ERK1/2 and p38 inhibitors indicated that the increase in 5-HT_2A _receptor activities was most likely dependent on IL-1β-induced alteration of MAPK pathways.

Organ culture has been used as a model to study alteration of G-protein coupled receptor in airway smooth muscle cells that associates with airway hyperresponsiveness. Moir and colleagues reported that organ culture of human intact bronchiole ring segments in serum free culture medium up to 6 days maintains its functional, biochemical and morphometric properties [19]. Smooth muscle-alpha-actin, myosin heavy chain isoform 1 expression, nonmuscle proteins, including total vinculin, beta-actin and nonmuscle myosin heavy chain were unchanged during the culture [19]. This agrees well with our previously report that organ culture of mouse tracheal segments in serum-free culture medium up to 16 days, the morphology and contractility of smooth muscle cells appeared to be maintained throughout the culture period [15]. By using the same model, we demonstrated that organ culture up-regulated bradykinin B_1 _and B_2 _receptors in the airway at transcriptional level [12,13]. The slight reduction of maximal contraction in response to 5-HT in organ culture in the present study could therefore be associated with the airway inflammation. This has been demonstrated by applying dexamethasone in the organ culture and seen that dexamethasone could abolish the reduced contractile response to 5-HT (unpublished data).

5-HT is released by neuroendocrine cells in the airways of animals and humans, its role in airway tone is not well understood [20]. During control conditions, 5-HT has been reported to show some effects in respiratory tissues by activation of different subtype receptors. It has been demonstrated that 5-HT_2A _receptor activation causes airway contraction *in-vivo *and *in-vitro*, and enhances effects of cholinergic nerve-mediated responses, whereas 5-HT_1A _receptor activation generally seems to be related to a relaxant effect [21-24]. In the present set-up, IL-1β induced a significant time- and concentration-dependent up-regulation of the contractile 5-HT response. Since ketanserin competitively antagonized the 5-HT-induced contraction with a pK_B _value 9.15 in accordance with 9.4 in mouse aorta [25], we could demonstrate that the contraction was mediated via 5-HT_2A _receptors. In analogy with a contractile role for 5-HT_2A _receptors in the airway, ketanserin, has been demonstrated to decrease ovalbumin-induced airway hyperresponsiveness in mouse [26] and to be beneficial in asthmatic subjects [27].

Release of epithelium-derived acetylcholine has been suggested to be an important factor in the development of the excessive tone often seen in murine and human airways following their exposure to different inflammatory mediators [4,28-30]. Accordingly, 5-HT is reported to induce an atropine sensitive bronchoconstriction in mice [18], an effect traditionally interpreted as mediated via cholinergic parasympathetic nerve fibers innervating the airway smooth muscles. However, recent findings suggest that the cholinergic contractile response to 5-HT in the mouse isolated trachea might depend on a non-neuronal source of acetylcholine, most likely the airway epithelium [4]. An atropine sensitive 5-HT response was also seen in the present set-up during fresh conditions, in accordance with a release of acetylcholine. In contrast, the 5-HT_2A _receptor-mediated contractions seen following long-term exposure to IL-1β appeared to be independent of both atropine and epithelium. Since ILs have been shown to increase ChAT activity and mRNA expression in the neuron cell culture [31,32], ChAT mRNA expression were quantified by real-time PCR in the present study, but no significant differences in ChAT mRNA expression between IL-1β and control groups were found. This together with that atropine failed to antagonize the 5-HT-induced contraction in the segments after IL-1β treatment, suggest an attenuated cholinergic signaling after IL-1β treatment regarding to 5-HT contractile response. Since no differences in carbachol-induced contraction between IL-1β and control groups were found, the mechanisms behind this is therefore most likely due to reduction of neural or non-neural acetylcholine production from surrounding tissues.

It is well known that 5-HT induced activation of 5-HT_2A _receptors results in intracellular phosphatidylinositol turnover and mobilization of Ca^2+ ^[33-35]. Studies have also demonstrated that 5-HT_2A _receptor-mediated contractions require activation of the intracellular MAPK ERK1/2 pathway [36,37]. The evaluation of acute effects of the MAPK inhibitors rather than their long-term effects is justified by the lack of transcriptional effects of IL-1β. In concert with this, the present study showed that acute inhibition of JNK, ERK1/2 or p38 activity decreased the contraction induced by 5-HT in fresh segments. However, in cultured segments the effect through p38 was decreased. When segments were treated with IL-1β, the effect through the ERK1/2 pathway was reduced concomitant with a strong effect through p38. The ability for IL-1β to interfere directly with MAPK pathways to raise the basic level of intracellular free Ca^2+ ^[38], can be the reason for the increased sensitivity to 5-HT stimulation.

## Conclusion

To summarize, the present study demonstrates that IL-1β can induce murine airway hyperresponsiveness, via a non-transcriptional up-regulation of 5-HT_2A _mediated contractile response through an alteration of the MAPK pathways. The fact that pro-inflammatory cytokines like IL-1β can alter airway contractile responses to contractile agents such as 5-HT, via interference with the intracellular MAPK signal transduction pathways, might provide a new concept for the treatment of asthma. Thus, a future therapeutic approach could be aimed to target the intracellular "on and off switch" for contractile airway receptors.

## Abbreviations

5-HT, 5-hydroxytryptamine; IL-1β, interleukin-1 beta; MAPK, mitogen-activated protein kinase; JNK, c-Jun N-terminal kinase; ERK1/2, extracellular signal-regulated kinase 1 and 2; TNF-α, tumor necrosis factor-alpha; DMEM, Dulbecco's Modified Eagle's Medium; PCR, polymerase chain reaction; ChAT, Choline acetyltransferase

## Competing interests

The author(s) declare that they have no competing interests.

## Authors' contributions

YZ carried out the experiments, analyzed the data, participated in the design of the study, drafted and revised the manuscript.

LOC and MA conceived of the study, participated in its design, helped to draft and revise the manuscript. All authors read and approved the final manuscript.
